# An Audience-friendly Medical Meeting: A Good Presentation and Chairpersonship

**DOI:** 10.31662/jmaj.2023-0219

**Published:** 2024-06-03

**Authors:** Shigeki Matsubara, Daisuke Matsubara

**Affiliations:** 1 Department of Obstetrics and Gynecology, Jichi Medical University, Tochigi, Japan; 2 Department of Obstetrics and Gynecology, Koga Red Cross Hospital, Koga, Japan; 3 Medical Examination Center, Ibaraki Western Medical Center, Chikusei, Japan; 4 Department of Pediatrics, Jichi Medical University, Tochigi, Japan

**Keywords:** Audience, audience-friendly, chairperson, meeting, presenter

## Abstract

The most important purpose of medical meetings is to share knowledge with the audience. Medical meetings should be audience-friendly. The presenter and chairperson play crucial roles in these meetings. We wish to put forward some personal proposals to make meetings audience-friendly. For the presenter, state the conclusion or significance first (in the case of case presentation), cite a fundamental article only, and do not skip reading in the summary slide. For the chairperson, be a timekeeper, stop the presentation when there are important mistakes, and choose a question that illustrates the significance of the study and thus interests the audience. All the meeting participants should understand this and support audience-friendly meetings.

## 1. Our Proposals

### The meeting should be more audience-friendly

After the pandemic, ordinary face-to-face medical meetings have resumed. Having a no-meeting period and then resumption helped us recognize some problems with medical meetings, namely, unfriendliness of presentations to audiences. Our conclusion of this opinion paper is that meetings should be more audience-friendly. A recommendation for making good “virtual” meetings in the COVID-19 era has been proposed ^(1)^. Although this recommendation involves many important messages that are applicable to conventional face-to-face meetings, to the best of our knowledge, there are few publications on how to conduct an audience-friendly meeting. The first author (SM) has written 529 PubMed-indexed papers. We have been participating in medical meetings as an audience, presenter, chairperson, and organizer. Taken together, we wish to make our proposals to conduct author-friendly meetings based on our experience.

### The meeting exists for whom? Proposals for a presenter and chairperson

Meetings involve four types of persons: audience, presenter, organizer, and chairperson. Meetings should be for all; however, the top priority is undoubtedly the audience. Thus, meeting is considered to have been successful if the audience has been able to grasp the message.

A successful meeting requires that all (the audience, presenter, organizer, and chairperson) have a common understanding that the meeting is for the audience. For simplicity, we describe our proposals for the two types of participants, the presenter and chairperson. If the remaining two, the audience and organizer, understand the fundamentals of our proposals, they will grasp the meaning of the behaviors and attitudes of the presenter and chairperson and thereby support them.

## 2. For Presenters

### Proposals for a presenter: General remark

We wish to make some proposals for a presenter, describing an obstetrics and gynecology case presentation as an example (Table 1). Almost all holds true for the original study presentation (not confined to the case presentation). We wrote some differences between case and original study presentation in Table 1. We refrain from stating basic matters, such as making clear slides with sans serif font ^(2)^.

**Table 1.** Proposals for an Audience-Friendly Meeting (for the Presenter).

1.	At the beginning:
	1) Do not read out COI when there is no COI. Just stating “No COI” in the slide is sufficient.^＊1-1)^
	2) State the conclusion. State the significance.^＊1-2)^
2.	During presentation:
	Do not cite many references. If one wishes, cite one. Rather, state “Search PubMed with X and Y,” if necessary.^＊2^
3.	At the end:
	1) Read the summary in an audience-friendly manner. Never end the presentation with “See the summary.”^＊3-1)^
	2) No need to make an Acknowledgment slide.^＊3-2)^
4.	In the question-and-answer portion:
	No need to state gratitude to the questioner.^＊4^

This is a personal proposal typically applicable to a case presentation but fundamentally holds for the presentation of original studies. ^＊1-1)^COI should be clearly stated if it exists, particularly in original studies with intervention. ^＊1-2)^In the presentation of an original study, the conclusion should not be stated at the beginning. Instead, the study question (hypothesis) should be stated. ^＊2^Remembering the cited article is impossible. One can remember keywords for PubMed search. ^＊3-1)^Summary is the essence of the presentation. The audience wishes to grasp it. ^＊3-2)^Persons greatly acknowledged should be listed as copresenters. ^＊4^One may state gratitude when the questioner makes a beneficial suggestion. Except for such cases, stating gratitude to each questioner is unnecessary as scientific meetings are places to ask questions. These are not rules but simple thoughts on how to create audience-friendly meetings.

### Good presentation

We will put forward proposals in the order that the presentation proceeds: from the beginning of the presentation to the question-and-answer portion (Table 1).

At the beginning, there is no need to read out any conflict of interest (COI); if so, state “no COI” in the slide. If there is COI, one must clearly explain it. A short statement regarding the medical significance should be provided at the beginning. For example: “uterine rupture usually manifests as an acute maternal shock. In this case, the omentum covered the rupture site and was therefore asymptomatic. Asymptomatic and, thus, unrecognized rupture can exist” ^(3)^. Stating this significance and the conclusion at the beginning relaxes the presenter and facilitates better understanding by the audience. This may also help avoid overrunning the allocated time (time overrun). At least, this avoids the situation whereby a presenter rushes to state the conclusion at the end of the presentation due to possible time overrun. This is an identical technique employed in case report publication ^(4)^.

Next, cite a fundamental article only. Nobody actually views all of them. If necessary and only when it is an extremely important article, cite only one with the first author’s name, publication year, and journal name. Or, for example, state the following: “PubMed search with ‘uterine rupture Matsubara’ provides full information and related articles.” The audience cannot remember Matsubara S. JOGR 2020; 46:1927 ^(3)^ but can remember “rupture Matsubara.”

At the end of the presentation, read the summary in a manner that the audience can grasp, preferably in a sentence-by-sentence manner. Thus, the summary should be concise. Never end the presentation with “Please see this summary slide” without reading it. Please do not state in the summary “We encountered uterine rupture”; every audience member understands this. Straightforwardly state your message: “Rupture is masked by the surrounding organ.”

An acknowledgment slide is not necessary, except when an acknowledged person has greatly contributed but, for some reason, cannot be a copresenter. Who is acknowledged does not matter, making a meeting different from a published article.

In the question-and-answer session, there is no need to state gratitude to each questioner. Some may disagree with this, saying that politeness is important. Then, the chairperson may state at the beginning of the session “To make the actual discussion time as long as possible, I asked the presenter to avoid an acknowledgment slide and expressing gratitude to the questioner.” This is modifiable in a case-by-case manner.

### What is an audience-friendly presentation? A summary

The audience wishes to know your message. Take sufficient time to explain your message while refraining from stating one pattern or customary expressions. Balance the former and latter.

## 3. For Chairpersons

### Proposal for a chairperson (Table 2)

First, be a timekeeper. A time overrun is the worst scenario; some audience members may have an airplane reservation. A session ending early does not mean an inactive session but indicates that the presentation satisfied all and thus ended early. The chairperson asking a question because no one else does usually causes time overrun. The chairperson should ask a question only when an extremely important message is not addressed. One should end early in preference to time overrun.

**Table 2.** Proposals for an Audience-Friendly Meeting (for the Chairperson).

1.	Be a timekeeper:
	・An early end does not matter.
	・If there are no questions, the chairperson need not question, except when extremely important issues remain unaddressed.
2.	Organize the presentation in a time-saving and audience-friendly manner:
	・Stop the presentation when there are mistakes that will shake the reliability of the presentation.
	・Ask whether important data are correct when doubtful.
3.	Select questions:
	Allocate a long time to questions regarding the significance of the study.
	Allocate a short time to questions regarding study details. Balance the two.

These are personal proposals. They are simple thoughts to make the presentation audience-friendly.

Second, when a chairperson recognizes mistakes, which will shake the reliability of the presentation, one must stop the presentation. For example, a less-experienced presenter may deliver a presentation with a different slide (previous or next). If the microphone condition is not good, a chairperson should demand to adjust it immediately. If misunderstandings by the presenter are proved after the presentation (e.g., stating 80 instead of 8 mg), the chairperson must point this out before the audience questions. This will save audiences from unnecessary confusion, and it also contributes to saving everybody’s time.

Third, choose questions. Similar to the article review, questions can be roughly divided into two: i) general (asking about medical significance/meaning) and ii) specific (asking about technical details) ^(5)^. Questioners can ask whatever they wish to know, irrespective of i) or ii). An extreme example of ii) is “Which antibody did you use, A or B?.” This may interest this questioner but nobody else. The chairperson must immediately determine whether the question regards i) or ii). Allocate long and short times for i) and ii), respectively, Or say “stop” to ii)-type questions. Saying “Stop” while maintaining a friendly atmosphere among the audience, presenter, and questioner requires knowledge, experience, and skills.

### What is an audience-friendly chairpersonship? A summary

The chairperson is not a person who introduces a presenter or says “thank you for listening” to the audience but a person who controls the session. How is the session proceeding and how deeply does the audience understand the presentation? This decides whether the chairperson should be silent or say something. The chairperson’s character does not decide this. A chairperson having performed a good job should be highly respected.

## 4. Purposes of Medical Meetings: Medical Meeting for Whom?

We understand that meetings have some purposes other than knowledge-sharing. Meetings (particularly face-to-face ones) facilitate friendliness among participants. This may nurture a cooperative study among them. Meetings enable younger generations to experience an academic atmosphere. The presenter will become self-confident in a step-by-step manner after experiencing delivering some presentations. The provider (scientific society and director/staff of the meeting) will accumulate knowledge regarding how to promote a good meeting. Thus, meetings nurture all (audience, presenter, and provider). However, the top priority of the medical meeting is to share knowledge with the audience.

We believe that Japanese medical meetings have been, and are, well organized. Presentations given by younger generations always encourage us. We love to participate in meetings. Hopefully, future presentations will be more audience-friendly.

## Article Information

### Conflicts of Interest

None

### Acknowledgement

This is our personal view and does not represent the opinions of any departments or medical societies with which we are associated.

### Author Contributions

S. Matsubara: Identification of the significance. Manuscript writing. D. Matsubara: Manuscript editing.

### Approval by Institutional Review Board (IRB)

Not applicable

### Patient Anonymity

Not applicable

### Informed Consent

Not applicable
